# Exploring the relaxation effects of *Coptis chinensis* and berberine on the lower esophageal sphincter: potential strategies for LES motility disorders

**DOI:** 10.1186/s12906-024-04720-x

**Published:** 2024-12-18

**Authors:** Wen-Harn Koh, Li-Wei Lin, Ting-I Lin, Ching-Wen Liu, Li-Ching Chang, I-Chun Lin, Ming-Shiang Wu, Ching-Chung Tsai

**Affiliations:** 1 Department of Pediatrics, E-Da Hospital, I-Shou University, No. 1, Yi-Da Road, Yan-Chao District, Kaohsiung City, 82445 Taiwan, R.O.C.; 2 School of Chinese Medicine for Post Baccalaureate, I-Shou University, No. 8, Yi-Da Road, Yan-Chao District, Kaohsiung City, 82445 Taiwan, R.O.C.; 3 Department of Senior Citizen Health Service and Management, Yuh-Ing Junior College of Health Care and Management, No. 15, Lane 420, Dachang 2nd Road, Kaohsiung City, 80776 Taiwan, R.O.C.; 4 School of Medicine for International Students, College of Medicine, I-Shou University, No. 8, Yi-Da Road, Yan-Chao District, Kaohsiung City, 82445 Taiwan, R.O.C.; 5 Department of Pediatrics, Kaohsiung Chang Gung Memorial Hospital, No. 123, Dapi Road, Niaosong District, Kaohsiung City, 83301 Taiwan, R.O.C.; 6 Department of Internal Medicine, National Taiwan University Hospital and College of Medicine, No. 7, Zhongshan S. Road, Zhongzheng District, Taipei City, 100225 Taiwan, R.O.C.

**Keywords:** *Coptis chinensis*, Berberine, Lower esophageal sphincter, Relaxation, Traditional Chinese medicine, Achalasia

## Abstract

**Background:**

Esophageal achalasia, a primary disorder impacting the lower esophageal sphincter (LES), presents symptoms such as dysphagia, regurgitation, chest pain, and weight loss. Traditional treatments, including calcium channel blockers and nitrates, offer limited relief, prompting exploration into alternative therapies. This study examines the efficacy of Traditional Chinese Medicine (TCM), focusing on *Coptis chinensis* (*C. chinensis*) and its principal component, berberine, for modulating LES relaxation, offering a new perspective on treatment possibilities.

**Methods:**

This research evaluated the impact of *C. chinensis* extract and berberine on the relaxation of LES contraction pre-induced by carbachol, observing the effects across different concentrations. We employed a series of inhibitors, including tetrodotoxin, ω-conotoxin GVIA, rolipram, vardenafil, KT5823, KT5720, NG-nitro-L-arginine, tetraethylammonium (TEA), apamine, iberiotoxin, and glibenclamide, to investigate the underlying mechanisms of berberine-induced LES relaxation.

**Results:**

Both *C. chinensis* extract and berberine induced significant, concentration-dependent relaxation of the LES. The relaxation effect of berberine was significantly reduced by TEA, indicating the involvement of potassium channels in this process.

**Conclusions:**

This study demonstrates that *C. chinensis* and berberine significantly promote LES relaxation, primarily through potassium channel activation. These findings provide a foundation for further investigation of these compounds’ potential therapeutic applications in esophageal motility disorders, such as achalasia.

**Graphical Abstract:**

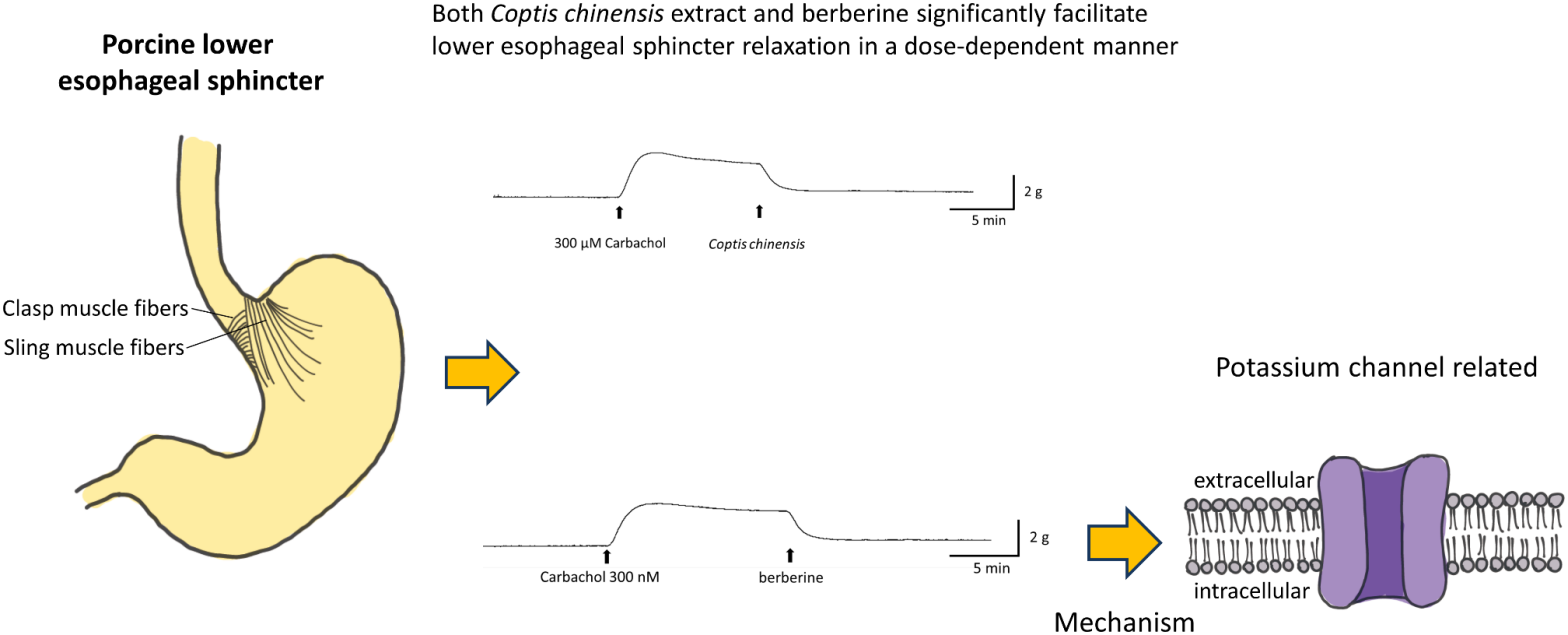

**Supplementary Information:**

The online version contains supplementary material available at 10.1186/s12906-024-04720-x.

## Introduction

Esophageal achalasia is a rare esophageal motility disorder marked by the loss of inhibitory neurons in the lower esophagus and its sphincter, resulting in no peristalsis and increased lower esophageal sphincter (LES) tone. This leads to the LES’s failure to relax properly, causing continuous contraction and food accumulation in the lower esophagus. Incidence rates of achalasia range from 1 to 12 per 100,000 annually [1]. Symptoms include progressive dysphagia, chest pain, regurgitation, and weight loss. Its exact cause remains unclear, possibly involving neuronal degeneration, viral infections, genetic factors, and autoimmune responses [2]. Diagnosis of achalasia typically involves a combination of three key tests: endoscopy, barium esophagram, and esophageal manometry. The therapeutic strategies for achalasia are diverse, encompassing pharmacological treatments, endoscopic procedures, and surgical approaches [3].

Early-stage achalasia is typically managed with pharmacological interventions, but options are limited. Calcium channel blockers such as nifedipine (10–30 mg sublingually before meals) and nitrates (5 mg before meals) are commonly prescribed for the treatment of achalasia [4]. However, these treatments often provide only partial relief and may have side effects, highlighting the need for more effective and better-tolerated options.

While current treatments for achalasia have limited efficacy, Traditional Chinese Medicine (TCM) offers potential alternatives that have not been fully explored. Among these, *Coptis chinensis* (*C. chinensis*), known as Chinese goldthread or Huang Lian in TCM, has a long history of use for gastrointestinal disorders. *C. chinensis* is particularly noteworthy for its high content of berberine, an isoquinoline alkaloid [5]. Berberine has shown promise in addressing various gastrointestinal issues, in addition to its diverse pharmacological properties including anti-adipogenic, anti-dyslipidemic, anti-cancer, and antimicrobial effects [6, 7].

Several studies have highlighted the potential of *C. chinensis* and berberine in addressing gastrointestinal disorders. For instance, *C. chinensis*, traditionally used in Chinese medicine for abdominal pain and diarrhea, have shown promising results in recent studies. Research has demonstrated its analgesic effects in a rat model of irritable bowel syndrome, potentially through modulation of serotonin levels and cholecystokinin expression in the colon [8]. Berberine, its primary active compound, effectively depresses gastrointestinal smooth muscle contraction in rats. It reduces contraction amplitude in isolated duodenum and gastric strips by inhibiting myosin light-chain kinase (MLCK). This mechanism may explain berberine’s antidiarrheal effects [9]. These findings suggest potential applications in disorders involving altered esophageal smooth muscle tone, such as achalasia.

Given the historical significance of *C. chinensis* in TCM and berberine’s demonstrated effects on smooth muscle, this study aims to investigate their potential efficacy in treating achalasia. We hypothesize that *C. chinensis* extract and berberine will induce significant relaxation of the LES, potentially through modulation of smooth muscle function, which may involve ion channels or other signaling pathways. This research could open new avenues for achalasia management, addressing current treatment gaps with natural compounds that may have fewer side effects than conventional pharmaceuticals. Our study aims to test this hypothesis and elucidate the underlying mechanisms of action.

## Materials and methods

### Materials acquisition and preservation

Pig samples for this study, averaging 110 kg, were sourced from slaughterhouses accredited by the Council of Agriculture, Executive Yuan (Taiwan). The LES specimens, including segments of the esophagus and stomach, came from pigs not bred for experimental purposes and were euthanized through electric stunning followed by exsanguination. For preservation, the Krebs-Henseleit solution used in this research contained 1.2 mM NaH_2_PO_4_, 4.7 mM KCl, 118 mM NaCl, 25 mM NaHCO_3_, 1.8 mM CaCl_2_, and 14 mM glucose, with a pH adjusted to 7.4. This solution was oxygenated for 15 min with 95% O_2_ and 5% CO_2_ prior to use. After procurement, the LES samples were immediately immersed in the chilled solution and swiftly transported to the laboratory, typically within 30 min, ensuring their physiological integrity. The study was exempt from review by the Institutional Animal Care and Use Committee at E-DA Hospital, as the porcine esophagus and stomach were classified as food products, not live animal parts, in accordance with relevant regulations.

### Preparation of *C. chinensis* Solution

The refined extract of the traditional Chinese herb *C. chinensis* was sourced from Kaiser Pharmaceutical Co., Ltd. (Product No.: 8010, Batch No.: E12025, Tainan, Taiwan). For experimental use, a solution was prepared by dissolving 30 mg of this extract in 1 ml of 20% ethanol.

### Evaluation of relaxation effects

#### Evaluation of *C. chinensis* solution on relaxation of carbachol-pre-induced contraction in the porcine LES

Porcine LES serves as a valuable model for studying the human LES due to anatomical and physiological similarities, including comparable muscle structure and contractile properties [10, 11]. After collecting the LES specimens, which comprise the sling and clasp muscles, the mucosal layer was carefully removed to reveal these underlying muscle structures. These muscles were dissected into strips measuring 1 cm x 0.5 cm, secured with surgical threads, and immersed in an organ bath containing 5 ml of Krebs-Henseleit buffer. The bath was maintained at a constant temperature of 37 °C and continuously oxygenated with a mixture of 95% O_2_ and 5% CO_2_. An isometric transducer (FORT10g; Grass Technologies, RI, USA) connected to an amplifier (Gould Instrument Systems, OH, USA) transmitted data to a dedicated computer system (BIOPAC Systems, CA, USA), ensuring a consistent muscle tension of 1.0 g. This setup allows for the study of LES function [10].

In this study, the LES muscle strips underwent an initial 30-minute equilibration, followed by induction of contraction using 1 µM carbachol, and subsequent washing with Krebs-Henseleit buffer. After another 30-minute equilibration period, a contraction induced by 300 nM carbachol was set as the 100% benchmark. This concentration was chosen based on preliminary studies testing a range of carbachol concentrations (10 nM − 100 µM). We selected 300 nM as it is above the EC50, to avoid overly strong contractions that might mask the relaxation effects of the test compounds.

Subsequently, the study evaluated the relaxation effects of 0.06, 0.24, and 0.6 g/L concentrations of *C. chinensis* on sling and clasp muscle strips. These were obtained by adding 10, 40, and 100 µL of the pre-prepared *C. chinensis* solution to the 5 ml Krebs-Henseleit buffer. These concentrations were determined through preliminary studies to provide a range of effects from minimal to maximal response.

#### Evaluation of the relaxation properties of berberine in the LES pre-induced by carbachol

This section aims to explore the effect of berberine, an active component of Huang Lian, on relaxing the LES. As described in the previous steps, following a second 30-minute equilibration period and the pre-induction of contraction with 300 nM carbachol, varying concentrations of berberine (10 µM, 30 µM, 100 µM, and 300 µM) were introduced. These concentrations were selected based on preliminary studies to cover a range from minimal to maximal relaxation effects. The relaxation response of the LES was then recorded. Berberine used in this study was obtained from Cayman Chemical (MI, USA).

#### Role of neuronal transmission in Berberine-Induced relaxation of the LES

This study investigates the role of neuronal transmission in the relaxation effect of berberine on the LES. Following previous method, the study utilized 1 µM tetrodotoxin (TTX), a specific neuronal sodium channel inhibitor, and 1 µM ω-Conotoxin GVIA (CTX), a neuronal calcium channel blocker, both of which were pre-added to the organ bath 15 min before introducing 50 µM berberine [10].

### Effect of cyclic adenosine monophosphate (cAMP) and cyclic guanosine monophosphate (cGMP) pathway modulators on berberine-induced relaxation of the LES

The study examines how rolipram (a specific phosphodiesterase 4 (PDE-4) inhibitor that increases cAMP levels) and vardenafil (a phosphodiesterase 5 (PDE-5) inhibitor that increases cGMP levels) might enhance the relaxation effect of berberine on the LES. Following previous method, either rolipram or vardenafil at a concentration of 1 µM was added to the organ bath 20 min prior to the introduction of 50 µM berberine [10].

### Influence of cAMP, cGMP, and nitric oxide (NO) on berberine-induced relaxation of the LES

To elucidate the roles of cAMP, cGMP, and NO in the relaxation mechanism of the LES induced by berberine, specific inhibitors were employed. These included 1 µM KT5720, targeting cAMP-dependent protein kinase (PKA); 1 µM KT5823, inhibiting cGMP-dependent protein kinase (PKG); and 100 µM NG-nitro-L-arginine (L-NNA), a nitric oxide synthase antagonist. L-NNA blocks NO production, which typically reduces cGMP levels, while KT5823 and KT5720 inhibit the downstream effects of cGMP and cAMP pathways, respectively, potentially reducing smooth muscle relaxation. Following previous method, these drugs were added to the system 30 min prior to the introduction of 50 µM berberine [10].

### Investigation of potassium channels in berberine-induced relaxation of the LES

This section aims to reveal the role of potassium channels in the relaxation mechanism of the LES induced by berberine. A series of channel blockers were used for this purpose: 1 mM tetraethylammonium (TEA), a non-specific potassium channel blocker; 100 nM apamine (targeting small-conductance calcium-activated potassium channels), 200 nM iberiotoxin (IbTX), targeting large-conductance calcium-activated potassium channels; and 10 µM glibenclamide (an ATP-sensitive potassium channel antagonist). Following previous method, these blockers were introduced into the system 30 min before the addition of 50 µM berberine [10, 12].

### Data analysis

The data are presented as mean values with the standard error of the mean (SEM). For statistical evaluation, either the Student’s t-test or one-way analysis of variance (ANOVA) followed by a Tukey post-hoc test were applied. The smallest sample size included in this study was four. A p-value of less than 0.05 was considered statistically significant in all instances. All statistical analyses were conducted using SPSS version 24 (IBM Corp., NY, USA). Additionally, EC50 values, indicating the concentration for a half-maximal effect, were calculated using GraphPad Prism 5 software.

## Results

### High-performance liquid chromatography (HPLC) analysis of *C. chinensis*

Supplemental Fig. 1 presents the characterization of *C. chinensis* extracts, obtained from Kaiser Pharmaceutical CO., Ltd., using HPLC. In this chromatogram, berberine was used as the reference compound, exhibiting a retention time of 10.48 min.

### Evaluation of the relaxation effect of *C. chinensis* on porcine LES following contraction pre-induced by carbachol

**Fig. 1:**
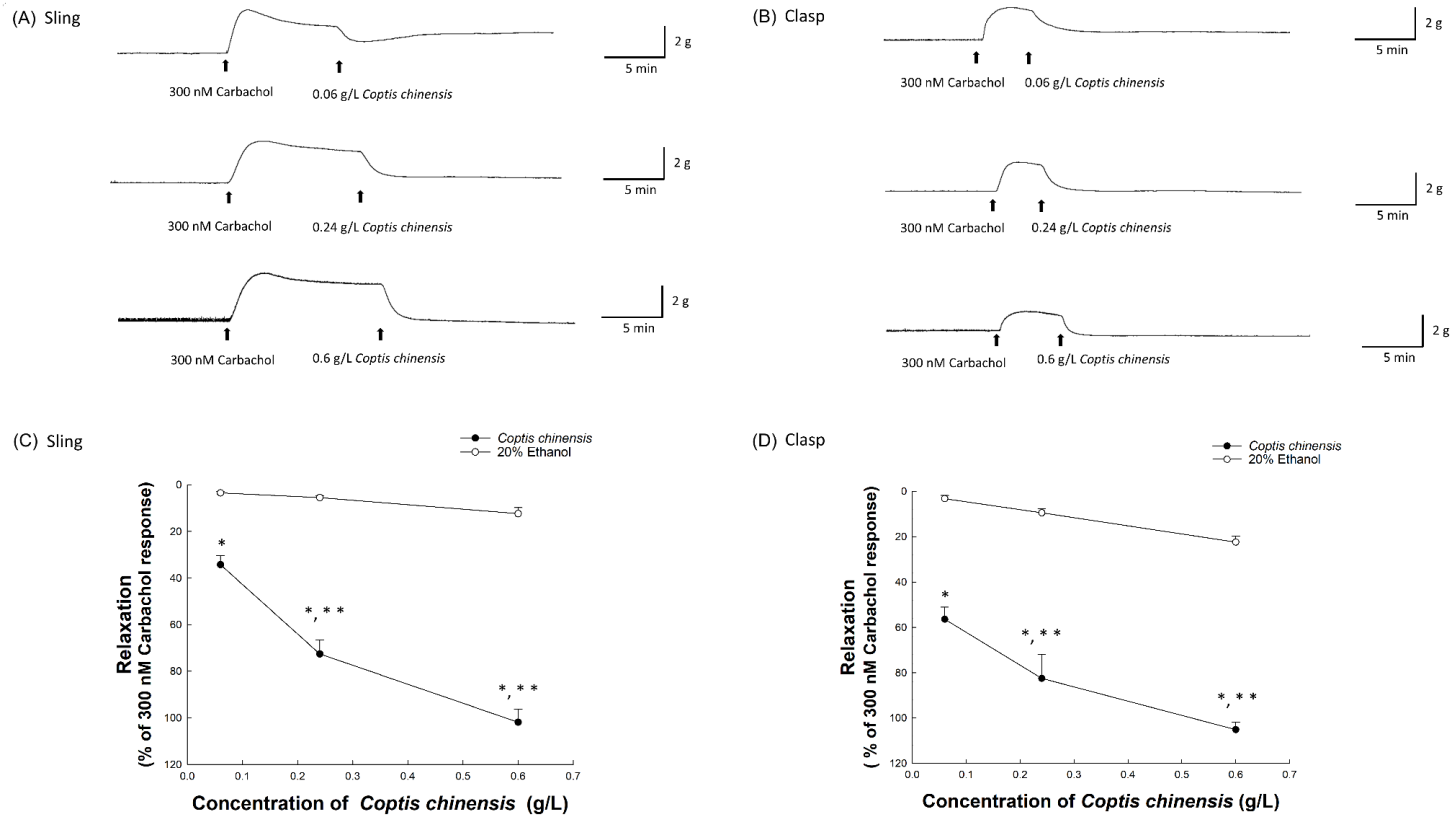
This figure showcases the relaxation effects of *Coptis chinensis* (*C. chinensis*) on porcine lower esophageal sphincter (LES) sling **(A)** and clasp **(B)** fibers following pre-induced contraction by 300 nM carbachol, with arrows indicating the subsequent addition of varying doses of *C. chinensis*. Panels C and D plot the dose-dependent relaxation of LES sling and clasp muscle fibers to *C. chinensis*, compared to the corresponding 20% ethanol control, quantified as percentage changes from the baseline established by 300 nM carbachol-induced contractions. Data are presented from ≥ 4 experiments for *C. chinensis* and ≥ 3 for ethanol, with SEM error bars. Statistical significance against the corresponding 20% ethanol control (*) and the relaxation response to 0.06 g/L *C. chinensis* (**) are indicated (*p* < 0.05)

Figure 1A and B depict characteristic tracings that demonstrate the relaxation effects of *C. chinensis* at concentrations of 0.06, 0.24, and 0.6 g/L on sling and clasp muscle strips, respectively, which relaxed in response to these different concentrations. *C. chinensis* induced dose-dependent relaxation in both sling and clasp muscle strips (Fig. 1C and D). Relaxation effects ranged from 34.25 to 101.84% in sling strips and 56.38–105.14% in clasp strips at concentrations between 0.06 and 0.6 g/L. These relaxation responses were significantly different from those induced by the respective solvent (all *p* < 0.05; *C. chinensis*: *n* ≥ 4, ethanol: *n* ≥ 3). Furthermore, the effects at 0.24 and 0.6 g/L of *C. chinensis* were significantly distinct compared to the 0.06 g/L concentration in both types of muscle strips (all *p* < 0.05, *n* ≥ 4).

### Dose-dependent effects of *C. chinensis* constituents berberine on LES relaxation

**Fig. 2:**
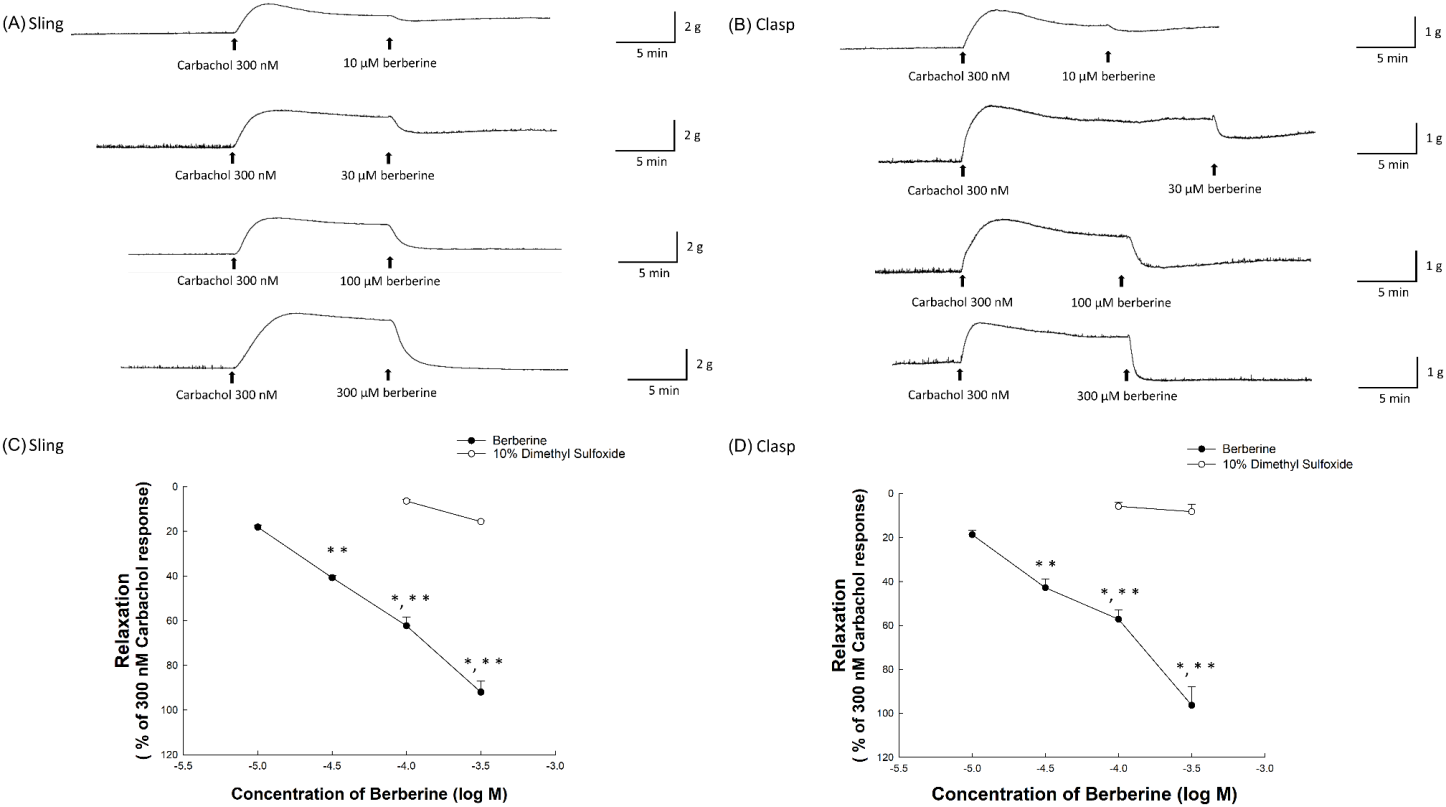
This figure depicts the relaxant effects of berberine on porcine lower esophageal sphincter (LES) sling **(A)** and clasp **(B)** fibers after contractions were pre-induced with 300 nM carbachol, highlighted by arrows indicating the sequential addition of carbachol and various doses of berberine. Panels C and D present a dose-dependent comparison of LES muscle relaxation to berberine against the corresponding 10% DMSO control, showcasing significant relaxation effects quantified as changes from the baseline established by carbachol-induced contractions. Data are presented from ≥ 4 experiments for both berberine and the corresponding 10% DMSO control, illustrated with SEM error bars. Statistically significant differences from the corresponding 10% DMSO control (*) and the relaxation effect induced by 10 µM berberine (**) are marked (*p* < 0.05)

In this study, we investigated the impact of berberine, a primary constituent of *C. chinensis*, on the relaxation response of the LES. The experiments were conducted after inducing LES contractions using 300 nM carbachol. Figure 2A and B illustrate the relaxation tracings in sling and clasp muscle strips, respectively, following treatment with representative berberine concentrations of 10, 30, 100 µM and 300 µM. These tracings demonstrate the progressive increase in relaxation effect with increasing berberine concentration, supporting our hypothesis of berberine’s dose-dependent impact on LES relaxation. Figure 2C quantifies the relaxation responses in the sling strips, showing relaxations of 18.12 ± 1.01% at 10 µM, 40.71 ± 1.04% at 30 µM, 62.24 ± 3.94% at 100 µM, and 91.96 ± 5.04% at 300 µM berberine. The 30 µM and 100 µM concentrations demonstrated significant differences compared to the corresponding solvent controls (both *p* < 0.05, *n* = 4). In the clasp strips, as shown in Fig. 2D, the relaxation responses were 18.12 ± 2.12% at 10 µM, 42.79 ± 3.87% at 30 µM, 57.13 ± 4.22% at 100 µM, and 96.28 ± 8.38% at 300 µM berberine. These 30 µM and 100 µM concentrations in the clasp strips also significantly differed from the corresponding solvent controls (both *p* < 0.05, *n* ≥ 4). Relaxation effects at 30, 100, and 300 µM were significantly greater than at 10 µM in both sling and clasp strips (all *p* < 0.05, *n* = 4). These findings support our hypothesis that berberine, a key constituent of *C. chinensis*, plays a significant role in LES relaxation, potentially contributing to its effects on gastrointestinal motility disorders. The EC_50_ value for berberine in sling strips was estimated to be approximately 50 µM (tested range: 1 µM to 1 mM), consistent with the concentration-dependent effects observed in both sling and clasp muscle strips. Therefore, we selected a concentration of 50 µM berberine for further investigation into its mechanism of LES relaxation. To ensure the robustness of our findings, we included appropriate controls in our experimental design. Estradiol, previously shown to induce marked relaxation in porcine LES strips [10], served as a positive control. As a negative control, we used coptisine, another component from *C. chinensis* extract, which our preliminary studies showed had no significant relaxation effect compared to the respective vehicle.

### Neural conduction’s influence on berberine-induced relaxation in porcine sling strips

**Fig. 3:**
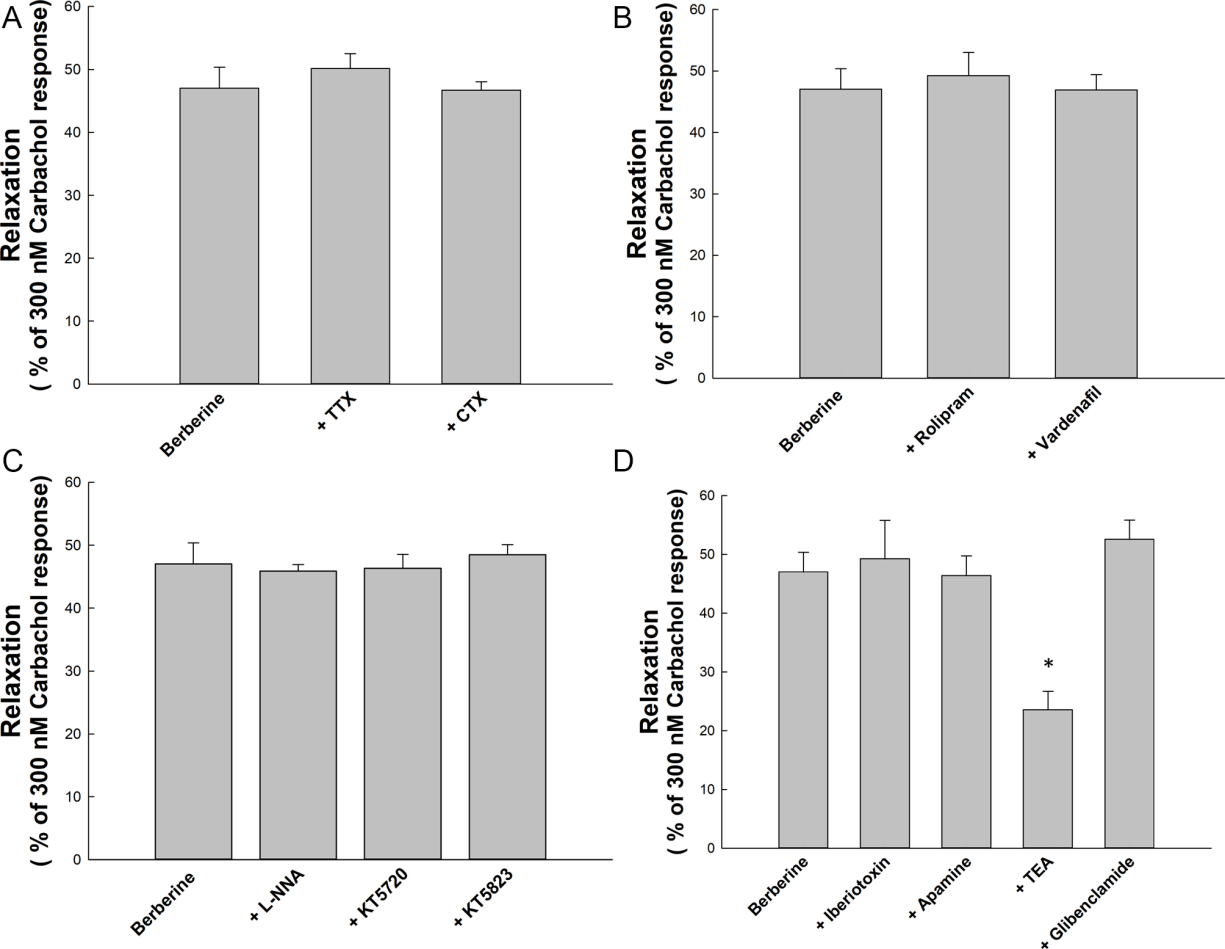
This figure summarizes the effects of various compounds on berberine-induced relaxation in porcine lower esophageal sphincter (LES). Panel A shows tetrodotoxin (TTX) and ω-conotoxin GVIA (CTX) had no significant effect on berberine relaxation (*p* > 0.05). Panel B indicates rolipram and vardenafil also did not significantly affect berberine relaxation (*p* > 0.05). Panel C demonstrates NG-nitro-L-arginine (L-NNA), KT5823, and KT5720 had no significant impact on relaxation (*p* > 0.05). Panel D reveals iberiotoxin (IbTX), apamine, and glibenclamide did not significantly alter berberine relaxation (*p* > 0.05), while tetraethylammonium (TEA) significantly inhibited relaxation (*p* < 0.05). Error bars represent SEM. A single asterisk (*) marks statistically significant difference from the relaxation effect induced by 50 µM berberine

Figure 3A shows that 1 µM TTX or 1 µM CTX did not significantly affect the relaxation of the sling strips induced by 50 µM berberine (*p* > 0.05, *n* = 4).

### Exploring the role of cAMP and cGMP in berberine-induced relaxation in porcine sling strips

Figure 3B demonstrates that 1 µM rolipram and 1 µM vardenafil had no enhancing effects on the relaxation of porcine sling strips induced by 50 µM berberine (*p* > 0.05, *n* = 4).

### The impact of cAMP, cGMP, and NO on berberine-induced relaxation in porcine sling strips

Figure 3C shows that 100 µM L-NNA, 1 µM KT5823, and 1 µM KT5720 did not inhibit the relaxation response in porcine sling strips induced by 50 µM berberine (*p* > 0.05, *n* = 4).

### The impact of potassium channels on *C. chinensis* and berberine-induced relaxation in porcine sling strips

Figure 3D shows that 100 nM apamine (*n* = 4), 200 nM IbTX (*n* = 5), and 10 µM glibenclamide (*n* = 4) did not inhibit the relaxation response of porcine sling strips induced by 50 µM berberine (*p* > 0.05). Conversely, 1 mM TEA significantly reduced this berberine-induced relaxation (*p* < 0.05, *n* = 4).

## Discussion

Our study demonstrates that *C. chinensis* significantly relaxes the LES. Previous research has shown that *C. chinensis* affects gastrointestinal tract motility and function. It demonstrates its effectiveness in alleviating visceral pain in irritable bowel syndrome [8], improving gastrointestinal function in combination with *Dolomiaea souliei* [13], and impacting immune health and metabolism in various processed forms [14]. Additionally, it enhances intestinal barrier function in treatments for ulcerative colitis [15].

Berberine, extracted from *C. chinensis*, is a bright yellow compound renowned for its antibacterial and anti-inflammatory properties. Historically utilized as a fabric dye [16], it has also shown potential in combatting atherosclerosis through mechanisms such as lipid regulation, reduction of blood pressure and blood sugar, inflammation control, and inhibition of vascular smooth muscle cell proliferation [17]. Furthermore, berberine demonstrates vasorelaxant and antiproliferative effects [18], highlighting its versatility and broad therapeutic potential.

The impact of berberine on gut microbiota is a major area of research, with significant implications for the management of diabetes, hyperlipidemia, atherosclerosis, and liver diseases [19]. Its role extends to metabolic regulation, underlining its potential in treating obesity and neurodegenerative diseases [20].

In addition to its relaxant impact on the LES observed in our study, berberine also significantly influences gastrointestinal motility. It modulates this motility by relaxing rat gastric fundus muscle through the inhibition of calcium entry [21], reduces the contractility of gastric intestinal smooth muscle [9], and demonstrates therapeutic effects on irritable bowel syndrome by inhibiting colonic smooth muscle neurotransmission [22]. Furthermore, berberine suppresses intestinal myoelectric activity and transit, potentially via opioid and α-adrenergic receptors [23], and obstructs both extracellular and intracellular Ca^2+^ flow in colon smooth muscle cells [24]. Among these effects, the antidiarrheal properties of berberine are particularly noteworthy, with a systematic review and meta-analysis affirming its efficacy and safety in treating diarrhea among both children and adults [25]. This evidence highlights berberine’s potential as a versatile treatment option for a range of gastrointestinal disorders.

Smooth muscle relaxation is a crucial physiological process mediated by multiple signaling pathways. To elucidate the mechanisms behind berberine-induced relaxation of porcine LES smooth muscle, we used various pharmacological agents targeting potential neural mediation and pathways involving cyclic nucleotides, nitric oxide, and potassium channels [26]. Our comprehensive approach to investigating the potential mechanisms provides valuable insights into berberine’s mode of action.

The assessment of potential neural mediation in berberine-induced relaxation of the porcine LES involved the use of TTX and CTX. The absence of inhibition with TTX and CTX is remarkable, suggesting that the relaxation induced by berberine in the porcine LES is not neurally mediated.

We employed rolipram and vardenafil, which are inhibitors of PDE-4 and PDE-5, respectively. By inhibiting these enzymes, rolipram and vardenafil increase the levels of cAMP and cGMP, potentially enhancing smooth muscle relaxation [7, 27]. However, these inhibitors did not enhance berberine’s relaxing effect on the porcine LES. Similarly, KT5720 and KT5823, which are selective inhibitors of cAMP and cGMP, had no impact on the berberine-induced relaxation in the LES. This indicates that the mechanisms of berberine-induced relaxation do not involve the cAMP, cGMP, or NO pathways [10]. Furthermore, the use of L-NNA, a competitive inhibitor of nitric oxide synthase, did not impede the berberine-induced relaxation of the porcine LES. This finding suggests that NO production is not associated with the relaxation induced by berberine.

Furthermore, we evaluated the role of potassium channels in the berberine-induced relaxation of the porcine LES using specific inhibitors. These included IbTX, apamine, TEA and glibenclamide. Notably, the blockade of potassium channels with TEA led to a significant reduction in the relaxation of porcine LES muscle [10, 12]. However, neither apamine, IbTX, nor glibenclamide significantly affected the berberine-induced relaxation in the porcine LES. These results suggest that the berberine-induced relaxation of the porcine LES is primarily mediated through potassium channels. Future research could further explore the exact molecular identity of the TEA-sensitive channels involved and investigate potential upstream signaling pathways that lead to the activation of these channels.

These findings underscore the distinct mechanism of action of berberine compared to conventional achalasia therapies. Conventional treatments such as nifedipine function by inhibiting calcium influx into smooth muscle cells, while nitrates work by increasing nitric oxide levels [1]. In contrast, berberine primarily induces LES relaxation via potassium channel activation, independent of cAMP, cGMP, and NO pathways. Consequently, berberine may offer an advantage over conventional treatments in terms of reduced systemic side effects.

Research highlights the effects of TCM and natural substances on the LES and esophageal function. The Modified *Xiaochaihu Decoction* enhances LES pressure and reduces ineffective swallowing, showing potential in treating gastroesophageal reflux disease [28]. *Arecae pericarpium* extracts, particularly arecoline, induce dose-dependent LES contractions [29]. Research indicates ginger does not alter LES resting pressure or esophageal contractions, but increases LES relaxation and reduces contraction velocity, potentially aiding gas expulsion [30]. 6-Gingerol, found in ginger, is known to increase LES tone [31]. *Curcumae longae Rhizoma* extract has been shown to lessen esophageal tissue damage and lower biochemical markers in acute reflux esophagitis [32]. Additionally, peppermint oil, a smooth muscle relaxant, effectively treats diffuse esophageal spasm [33]. These findings enhance our understanding of traditional medicines’ impact on LES motility.

Our findings on *C. chinensis* and berberine’s relaxant effects on the LES offer new perspectives for treating gastrointestinal disorders, bridging TCM with modern pharmacology. The identified potassium channel-mediated relaxation mechanism suggests potential for more targeted therapies. While potassium channel modulators are not yet widely used for gastrointestinal disorders, research has shown their promise in cardiovascular and neurological conditions [34–36]. This mechanism may have applications in other smooth muscle disorders [37] and could lead to personalized treatments for LES disorders, opening new directions in gastrointestinal medicine research.

Our pilot study provides valuable initial insights into the potential of *C. chinensis* and berberine for treating LES disorders. However, our research primarily relies on ex vivo porcine LES models, which limits full understanding of the compounds’ effects. To address these limitations and enhance the study’s impact, we propose incorporating in vitro cell line models and conducting animal studies. These additions will provide crucial insights into molecular mechanisms, toxicity, pharmacokinetics/pharmacodynamics, and safety profiles. Clinical trials are essential to confirm the relaxant effects on human LES and assess efficacy in treating LES motility disorders. Future research should prioritize optimizing dosage and administration methods, evaluating long-term safety and efficacy, exploring effects on other gastrointestinal motility disorders, and investigating potential synergies. Additionally, further studies could explore the effects of other compounds within *C. chinensis* and their potential contributions to LES relaxation and underlying mechanisms. By addressing these areas, we can work towards developing improved treatments for gastrointestinal motility disorders, offering alternative hope to patients with conditions like achalasia.

## Conclusion

This study underscores the significant relaxation effects exerted by both *C. chinensis* and its constituent, berberine, on the LES. The observed relaxant effect of berberine is likely mediated through potassium channels, which suggests a potential mechanism for the action of these agents. These findings pave the way for further research into the development of targeted treatments for gastrointestinal motility disorders, such as achalasia, based on these specific compounds and their demonstrated mechanisms of action.

## Electronic supplementary material

Below is the link to the electronic supplementary material.

Supplementary Material 1: Fig. 1. High-Performance Liquid Chromatography (HPLC) profile of berberine hydrochloride and *Coptis chinensis* (*C. chinensis*). Panel A depicts the HPLC trace of a standard berberine hydrochloride solution, with a pronounced peak at a retention time of 10.48 min, corresponding to 60 ppm of berberine hydrochloride. Panel B illustrates the HPLC trace of a *C. chinensis* sample, displaying multiple peaks. Notably, a peak at the same retention time of 10.48 min aligns with the berberine hydrochloride standard, suggesting the presence of this compound in the *C. chinensis* sample.

Supplementary Material 2

## Data Availability

All relevant data are included within the manuscript and are available from the corresponding author on reasonable request.

## Abbreviations

LES: Lower esophageal sphincter
TCM: Traditional Chinese Medicine
MLCK: Myosin light-chain kinase
HPLC: High-performance liquid chromatography
TTX: Tetrodotoxin
CTX: ω-Conotoxin GVIA
cAMP: Cyclic adenosine monophosphate
cGMP: Cyclic guanosine monophosphate
NO: Nitric oxide
PDE-4: Phosphodiesterase 4
PDE-5: Phosphodiesterase 5
PKA: cAMP-dependent protein kinase
PKG: cGMP-dependent protein kinase
TEA: Tetraethylammonium
IbTX: Iberiotoxin
L-NNA: NG-nitro-L-arginine
SEM: Standard error of the mean
ANOVA: One-way analysis of variance
