# Supplementary figures and images for: Exploring the relaxation effects of Coptis chinensis and berberine on the lower esophageal sphincter: potential strategies for LES motility disorders
Source: BMC Complement Med Ther. 2024 Dec 18;24:417. doi: 10.1186/s12906-024-04720-x (PMC11658121; doi:10.1186/s12906-024-04720-x)

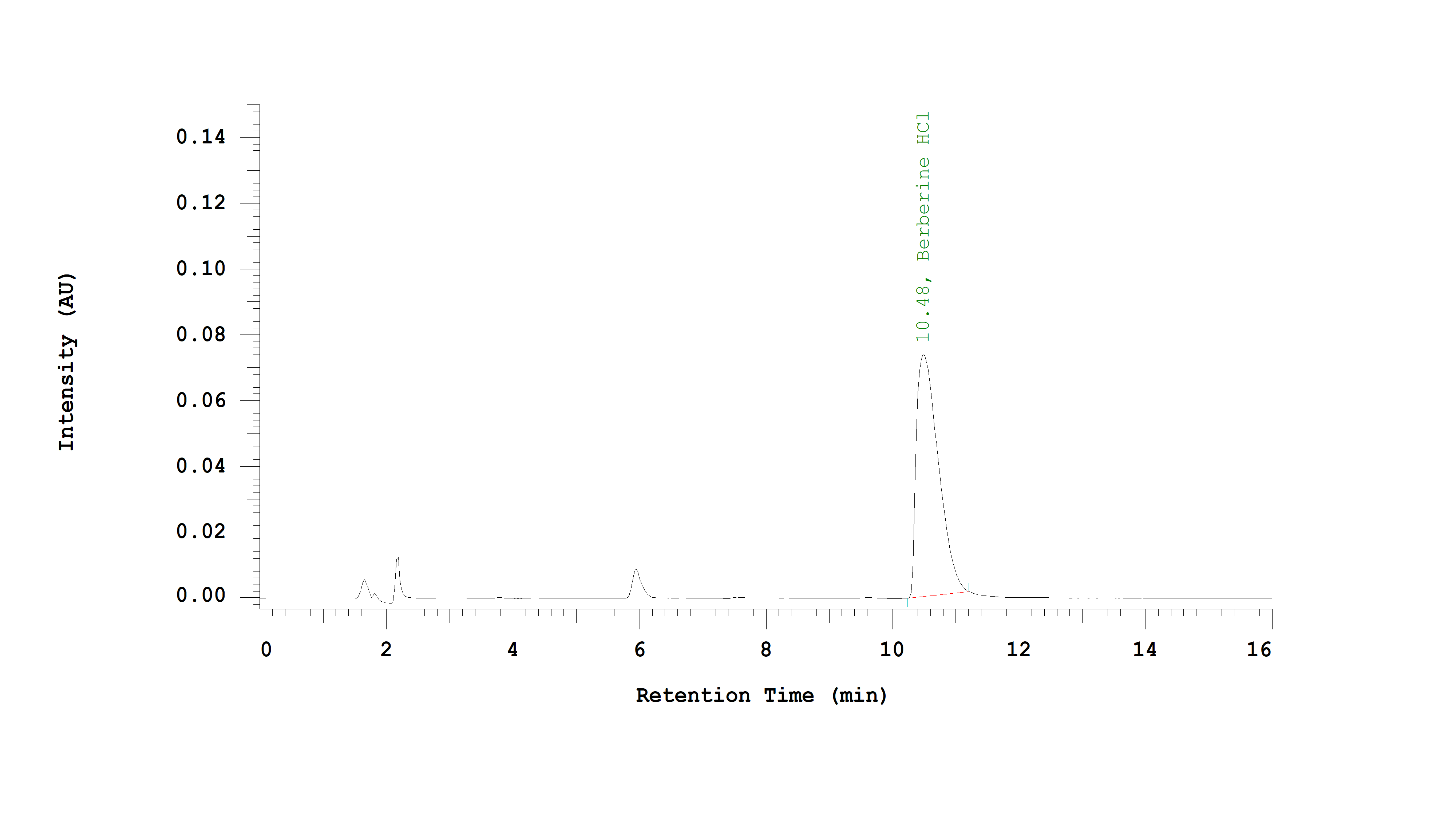

Supplement: Supplementary file 1 — Supplementary Material 1: Fig. 1. High-Performance Liquid Chromatography (HPLC) profile of berberine hydrochloride and Coptis chinensis (C. chinensis). Panel A depicts the HPLC trace of a standard berberine hydrochloride solution, with a pronounced peak at a retention time of 10.48 min, corresponding to 60 ppm of berberine hydrochloride. Panel B illustrates the HPLC trace of a C. chinensis sample, displaying multiple peaks. Notably, a peak at the same retention time of 10.48 min aligns with the berberine hydrochloride standard, suggesting the presence of this compound in the C. chinensis sample. [file 12906_2024_4720_MOESM1_ESM.tif]

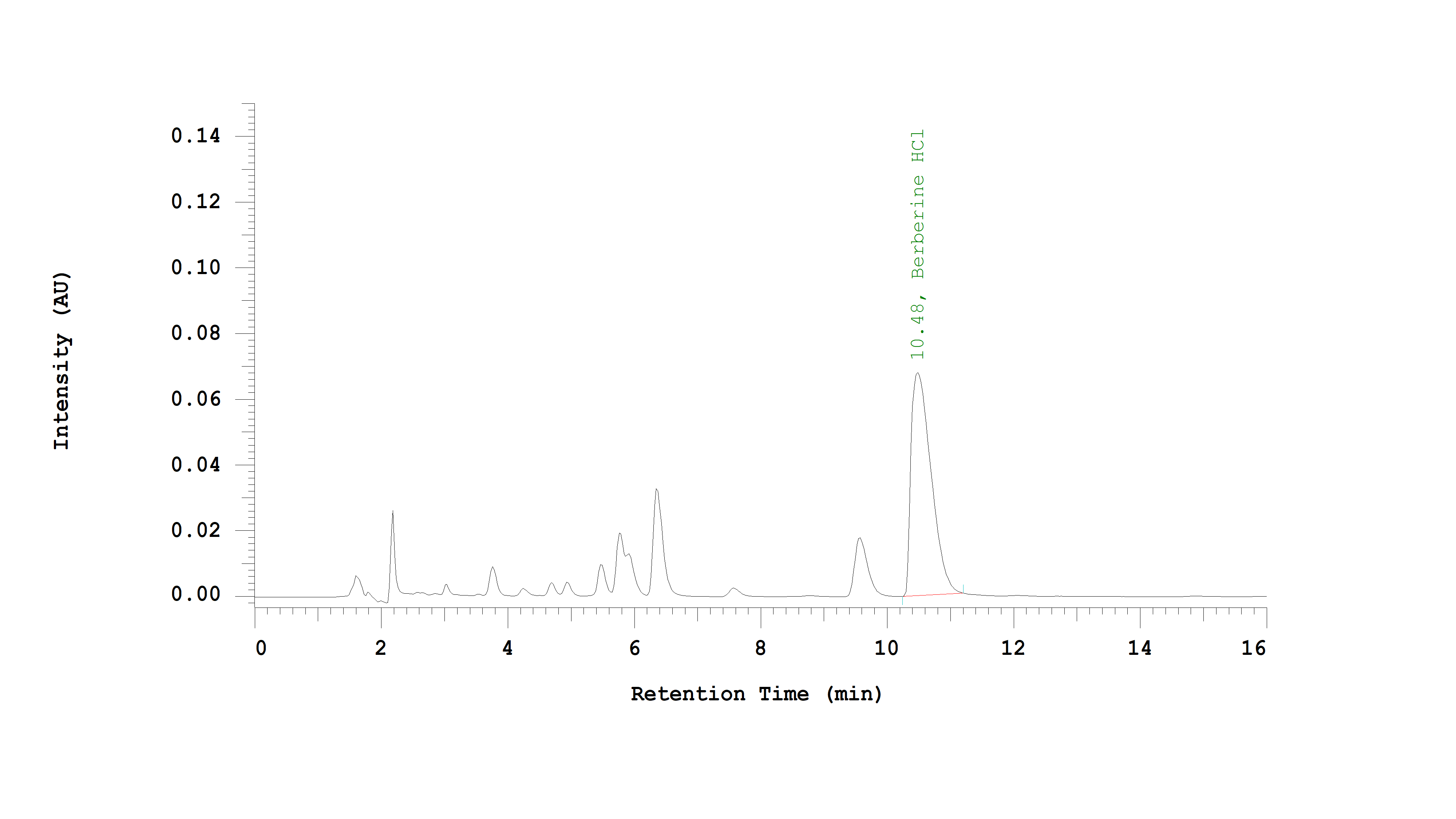

Supplement: Supplementary file 2 — Supplementary Material 2 [file 12906_2024_4720_MOESM2_ESM.tif]
